# Advancements and Challenges in Hydrogel Engineering for Regenerative Medicine

**DOI:** 10.3390/gels10040238

**Published:** 2024-03-30

**Authors:** Hossein Omidian, Sumana Dey Chowdhury, Renae L. Wilson

**Affiliations:** Barry and Judy Silverman College of Pharmacy, Nova Southeastern University, Fort Lauderdale, FL 33328, USA; sd2236@mynsu.nova.edu (S.D.C.); rw1273@mynsu.nova.edu (R.L.W.)

**Keywords:** hydrogel technology, tissue engineering, regenerative medicine, biofabrication, stem cell differentiation

## Abstract

This manuscript covers the latest advancements and persisting challenges in the domain of tissue engineering, with a focus on the development and engineering of hydrogel scaffolds. It highlights the critical role of these scaffolds in emulating the native tissue environment, thereby providing a supportive matrix for cell growth, tissue integration, and reducing adverse reactions. Despite significant progress, this manuscript emphasizes the ongoing struggle to achieve an optimal balance between biocompatibility, biodegradability, and mechanical stability, crucial for clinical success. It also explores the integration of cutting-edge technologies like 3D bioprinting and biofabrication in constructing complex tissue structures, alongside innovative materials and techniques aimed at enhancing tissue growth and functionality. Through a detailed examination of these efforts, the manuscript sheds light on the potential of hydrogels in advancing regenerative medicine and the necessity for multidisciplinary collaboration to navigate the challenges ahead.

## 1. Introduction

The field of tissue engineering is driven by the goal of addressing a broad spectrum of clinical challenges, particularly those involving the replication of the body’s native tissue environments. Central to this endeavor are hydrogel scaffolds, recognized for their ability to closely emulate the biomechanical and structural characteristics of natural tissues. These scaffolds provide an optimal environment that supports tissue integration and stem cell development while minimizing potential adverse reactions [1,2,3,4,5,6,7]. Achieving a harmonious balance between the biocompatibility, biodegradability, and mechanical strength of these scaffolds is a significant challenge but is pivotal for their successful clinical application [8,9,10,11]. The development of materials that are both bioactive and biodegradable poses particular difficulties in the regeneration of complex tissues, such as those requiring vascularization [8]. Furthermore, the engineering of scaffolds that accurately replicate the anatomical shapes and structures vital for effective tissue regeneration introduces additional design and fabrication complexities [12].

Advancements in creating supportive three-dimensional microenvironments have been instrumental in promoting cell growth and facilitating interactions within engineered tissues. However, optimizing bioinks for 3D bioprinting has highlighted some limitations inherent in traditional scaffold-based approaches [10,13,14,15,16]. The overarching aim of enhancing cell attachment, proliferation, and vascularization highlights the intricate balance of factors necessary for the success of tissue-engineered constructs [17]. The integration of 3D printing and biofabrication techniques has transformed the ability to create complex structures and precisely fabricate tissues [18,19,20]. Ongoing research into new materials and methodologies seeks to address the dynamic challenges faced in regenerative medicine [21,22].

Critical to the functionality and repair of tissues are improvements in the mechanical properties of materials, precise control over scaffold erosion for drug delivery, and the enhancement of nutrient and oxygen transfer [4,23,24]. The diverse and complex nature of clinical conditions in tissue engineering necessitates customized solutions for different tissue types, further illustrating the field’s challenges [25,26,27,28]. Tissue engineering is thus defined by its multifaceted obstacles, ranging from the development of hydrogel scaffolds with specific biomechanical and biochemical properties to crafting organ-specific solutions. This underscores the importance of continued innovative research and a multidisciplinary approach to overcome the hurdles in tissue regeneration and repair.

## 2. Engineering Advanced Hydrogels for Biomedical Applications

Hydrogels are increasingly recognized in the field of biomedical engineering for their remarkable similarity to the natural extracellular matrix. This similarity includes their ability to retain water, their biocompatibility, their tunable mechanical properties, and their capability to encapsulate both cells and bioactive molecules. These features make hydrogels invaluable tools in regenerative medicine, drug delivery, and tissue engineering. To enhance these already beneficial properties, researchers have been working on modifying the composition and structure of hydrogels. This involves integrating various polymers such as polyethylene glycol (PEG), hyaluronic acid, gelatin, and alginate. These polymers are chosen for their ability to confer essential characteristics for medical applications, such as flexibility and responsiveness to environmental stimuli [3,13,29]. The integration of nanostructures, like noble metal nanoparticles or silicon–phosphorus nanosheets, into hydrogels further boosts their mechanical strength and bioactive properties, broadening their application scope [1,2,8].

Advancements in creating multicomponent bioinks and complex polymer networks are crucial for developing hydrogels that can be precisely tailored for specific biomedical applications [4,15]. By optimizing the hydrogels’ structural and mechanical properties through adjustments in concentration and crosslinking methods, scientists can refine these materials to suit particular tissues or organs [1,14,30]. Moreover, enhanced biocompatibility and functionality is achieved by designing macroporous gel scaffolds that promote cell adhesion and proliferation. The inclusion of bioactive molecules or nanoparticles within these scaffolds improves their stability and functionality, making them even more effective for medical use [22,29,31].

The interdisciplinary nature of hydrogel development, which encompasses materials science, biology, and engineering, is key to customizing hydrogels for diverse applications. This customization ranges from creating hydrogels specifically designed for tissue engineering and bioprinting that support the growth and differentiation of certain cell types to utilizing 3D bioprinting techniques for assembling complex tissue constructs [8,20]. Additionally, innovative fabrication techniques such as rapid-cooling cryopreservation and replica molding play critical roles in ensuring cell viability and facilitating the rapid prototyping of tissue constructs [32].

The ongoing innovations in hydrogel fabrication and design are paving the way for the next generation of hydrogels. Novel 3D printing and bioprinting techniques enable the creation of intricate structures with unparalleled precision [33,34]. Moreover, developing hydrogels that can respond to environmental stimuli promises the advent of dynamic, responsive tissue scaffolds [12,24]. As the field of hydrogel research continues to evolve, the advancements in polymer chemistry, nanotechnology, and bioengineering are expanding their applications in biomedical science. This progress addresses current challenges in tissue engineering, regenerative medicine, and drug delivery, opening new therapeutic possibilities for improving patient care.

## 3. Common Hydrogel Materials in Regenerative Medicine

In tissue engineering, hydrogel research is primarily focused on developing materials that are not only compatible with biological systems but also mechanically robust and functionally akin to the natural extracellular matrix (ECM). This compatibility is crucial for promoting enhanced cell interactions and facilitating the integration of cells into tissues.

Polyethylene glycol (PEG)-based hydrogels are renowned for their versatility and the broad scope for customization they offer. Examples include a four-arm PEG [13,35], an engineered PEG variant sensitive to proteases and functionalized with bioligands [29], and derivatives like polyethylene glycol diacrylate (PEGDA) when combined with gelatin methacryloyl (GelMA) for bone regeneration [8]. These examples highlight PEG’s wide-ranging adaptability and its suitability for various tissue engineering applications.

Natural polymer-based hydrogels, crafted from materials such as fibrin–agarose [1], methacrylated hyaluronic acid, blends of alginate and gelatin [3], different forms of alginate [18,36,37], and collagen-based systems [20,38,39], are esteemed for their biocompatibility and their close mimicry of the ECM.

Gelatin methacryloyl (GelMA) and its derivatives are distinguished by their biocompatibility and the capacity to precisely adjust their mechanical properties. Their extensive application is due to their beneficial impact on cellular activities, such as matrix metalloprotease expression [14], and their use in regenerating bone tissues [8], as well as various other tissues like the intervertebral disc, nerve, and soft tissues [40,41,42,43,44].

Alginate-based hydrogels are appreciated for their straightforward gelation process and porous structure, making them suitable for cryopreservation [32], the creation of porous constructs [36], injectable scaffolds [45], and the regeneration of bone, cartilage, and neural tissues [46,47].

Composite and functionalized hydrogels, incorporating elements like noble metal nanoparticles [2], graphene oxide [22], and polymeric blends such as chitosan/gelatin for neo-vessel growth [17] and polyacrylamide/graphene oxide/gelatin/sodium alginate (PAM/GO/Gel/SA) for nerve regeneration [48], are engineered to merge mechanical durability and electroconductivity with biological functionality for precise applications.

Hybrid and synthetic–natural hydrogels combine the advantages of synthetic polymers like PEG with natural ones, such as collagen [39], to optimize both mechanical strength and biological properties for targeted tissue engineering endeavors.

Extracellular matrix (ECM)-derived and decellularized hydrogels, utilizing materials from sources like the porcine urinary bladder (pUBM) [9,49] and decellularized tissues such as the small intestine submucosa (SISMA) [22], offer a biomimetic environment conducive to cell attachment, proliferation, and differentiation.

For specialized applications, hydrogels are tailored for specific regenerative purposes, from cartilage regeneration using hyaluronic acid loaded with kartogenin and synthetic melanin nanoparticles [50] to neural applications with electrospun poly(lactide-co-epsilon-caprolactone) (PLCL) [51], and spinal cord regeneration using chitosan nanocellulose loaded with adipose-derived stem cells [52].

Supramolecular and nanofiber hydrogels [53] represent a shift towards materials that not only support cell adhesion and proliferation but also enhance scaffold bio-functionality through a bioinspired design strategy, marking a significant advancement in the field of tissue engineering.

## 4. Trends in Hydrogel Research for Regenerative Medicine

Hydrogels are increasingly tailored to modulate cellular behaviors and facilitate the regeneration of specific tissues. This customization is achieved by incorporating bioactive molecules such as bioligands [29], growth factors [42,54], and bioactive peptides [49,55], which trigger specific cellular responses conducive to processes like osteogenesis, angiogenesis [8], and cell differentiation [35,56,57]. Such strategic incorporations are designed to meet the regenerative requirements of targeted tissues.

Progress in this field is also driven by the adoption of advanced fabrication techniques, including 3D bioprinting [15,22,44,58], crosslinking [30,33], and other innovative approaches [4,35,41]. These techniques enable the creation of hydrogel scaffolds with precise geometries and functionalities, allowing for the hydrogels’ customization across various tissue types, including vascularized bone tissue [8], cardiac tissues [59], and specific regeneration efforts in the spinal cord [60] and urethra [58].

Hybrid and composite hydrogels, which combine natural and synthetic polymers [15], nanoparticles [2], and a variety of nanomaterials [8,22,61], are developed to achieve optimal mechanical, electrical, and biological properties. By integrating both organic and inorganic components [62,63], these hydrogels are engineered to enhance tissue support, offering improved strength, conductivity, and tailored biological responses.

Dynamic and responsive hydrogel systems are at the forefront of current research, focusing on injectable [21,24,28,61], thermosensitive [28,61,64,65], and dynamically responsive hydrogels [5]. Such systems are designed to provide environments that can adapt to biological conditions or respond to them, emphasizing minimally invasive and patient-specific treatment approaches.

The architectural design of hydrogel scaffolds, including porosity [25,36,66] and oxygenation [25], plays a crucial role in boosting cell viability and ensuring their seamless integration with host tissues. Advanced imaging [67] and monitoring techniques are employed to non-invasively assess scaffold performance, verifying their effectiveness in supporting tissue development.

Research in stem cell engineering within the hydrogel domain [68,69,70,71,72] highlights the potential of these materials in stem cell-based therapies. Tailoring hydrogel properties to support the encapsulation and differentiation of various cells, such as chondrocytes, multipotent stromal cells, and adipose-derived stem cells, addresses specialized needs in cartilage [26,37], bone [25], and neural regeneration [52]. This approach reflects a concerted effort to develop materials that cater to the unique regenerative requirements of different tissues.

Efforts to mechanically reinforce hydrogel scaffolds [7,50] and explore innovative crosslinking techniques [24,30] aim to produce constructs that can endure physiological stresses while fostering tissue development. This includes the integration of conductive materials [73] and the application of electrical stimulation [51,73] to boost regeneration, especially in neural and vascular tissues. Such developments mark significant steps in crafting hydrogels specifically designed for regenerative medicine applications.

## 5. Advancements in Hydrogel Applications for Tissue Engineering

Recent studies have highlighted the importance of hydrogel properties in tissue engineering and regenerative medicine. One study using a four-arm PEG hydrogel found that the softness of the hydrogel plays a crucial role in the growth of mouse ovarian follicles in vitro, emphasizing that specific softness levels can enhance oocyte maturation and development [13]. Another investigation focused on fibrin–agarose hydrogels, which were crosslinked with glutaraldehyde (GA), discovered that crosslinking improves the hydrogels’ structural and biomechanical qualities, particularly noting that hydrogels with 0.25% GA show promising features for tissue engineering applications [1].

Further research into protease-responsive PEG hydrogels, which are functionalized with bioligands, showed their effectiveness in supporting epithelial morphogenesis in 3D cultures, thereby aiding tissue regeneration [29]. One study on the role of macropores in hydrogels indicated that these structures significantly improve cell adhesion, proliferation, and extracellular matrix (ECM) deposition, enhancing the performance of scaffolds used in tissue engineering [31].

An examination of gelatin methacryloyl (GelMA) hydrogels revealed that the concentration of GelMA affects matrix metalloprotease (MMP) expression in adipose-derived stem cells (ASCs), suggesting that GelMA’s mechanics influence the cells’ ability to remodel the ECM [14]. Research comparing photocrosslinked hydrogels made from blends of methacrylated hyaluronic acid, alginate, and gelatin concluded that these materials exhibit excellent biocompatibility and promote cell adhesion [3].

Studies on metal nanoparticle–hydrogel composites have demonstrated their potential in enhancing both the physical and biological properties of scaffolds for tissue engineering [2]. Additionally, advances in controlling the mechanical properties of hydrogels were discussed, showcasing techniques for fine-tuning these materials for various applications [4]. The development of core–shell alginate hydrogels encapsulating stem cells for cryopreservation was highlighted, showing the potential for improving storage and distribution of stem cell-laden microcapsules [32].

A study on GelMA-PEGDA hydrogels with silicon–phosphorus nanosheets has shown promising results for vascularized bone regeneration, indicating enhanced osteogenesis and angiogenesis [8]. A review of multicomponent hydrogel-based bioinks for extrusion 3D bioprinting has identified suitable bioinks for different needs, emphasizing the ongoing challenges in 3D bioprinting [15].

Given chitosan’s biocompatibility, antibacterial properties, and biodegradability, the potential of 3D-printed chitosan (CS) scaffolds in biomedical applications has been highlighted. One review highlights the advancement of 3D printing in overcoming traditional manufacturing limitations by allowing precise control over scaffold properties, which is crucial for tissue engineering and regenerative medicine. The review serves as a roadmap for further optimizing CS-based constructs for clinical use, integrating this understanding into the broader context of regenerative medicine and tissue engineering [74].

**Figure 1:**
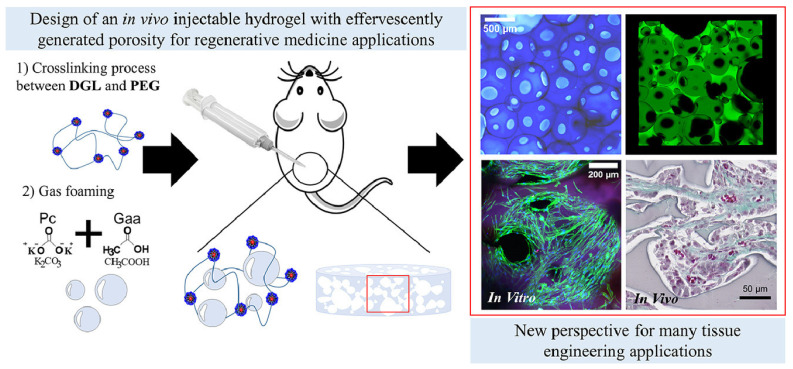
Schematic representation of developing effervescent porous hydrogels (EPHs) for tissue engineering applications [24].

Research on PEG hydrogels with lysine dendrimers has developed an injectable hydrogel that forms porosity in situ, supporting extensive neovascularization and tissue remodeling post injection (Figure 1) [24]. Alginate hydrogel with gelatin beads was found to enhance nutrient and oxygen transfer, leading to improved cell proliferation and function in porous scaffolds [36].

Studies on photocrosslinked PEG bilayer hydrogels employing bio-origami techniques for self-folding scaffolds have demonstrated their viability and functionality in cells over eight weeks [12]. Studies on the impact of crosslinker length on hydrogel formation dynamics have shown the ability to tailor hydrogel properties and polymerization speed [30].

Further research includes the 3D printing of cell-laden collagen hydrogel structures for regenerative medicine, which allows for uniform cell seeding and long-term viability [20]. The use of PDMS from laser-etched acrylic for making molds has enabled the control of tissue morphology in engineered cardiac tissues [59]. A keratin-based hydrogel for 3D-printed scaffolds has shown superior mechanical properties and biocompatibility [33]. Additionally, the overview of laser-based degradation of hydrogels has enabled high-resolution scaffold customization [75].

Research has employed a methacryloyl-modified decellularized small intestine submucosa (SISMA) with graphene oxide to develop a multi-responsive hydrogel for 3D bioprinting, enhancing structural characteristics and electrical conductivity for stem cell viability [22]. Furthermore, the use of a sodium alginate hydrogel in a novel multi-nozzle bioprinting system has confirmed the feasibility of free-form structures, showcasing the versatility of hydrogels in advanced tissue engineering and regenerative medicine applications [18].

## 6. Stem Cell Engineering and Differentiation

**Figure 2:**
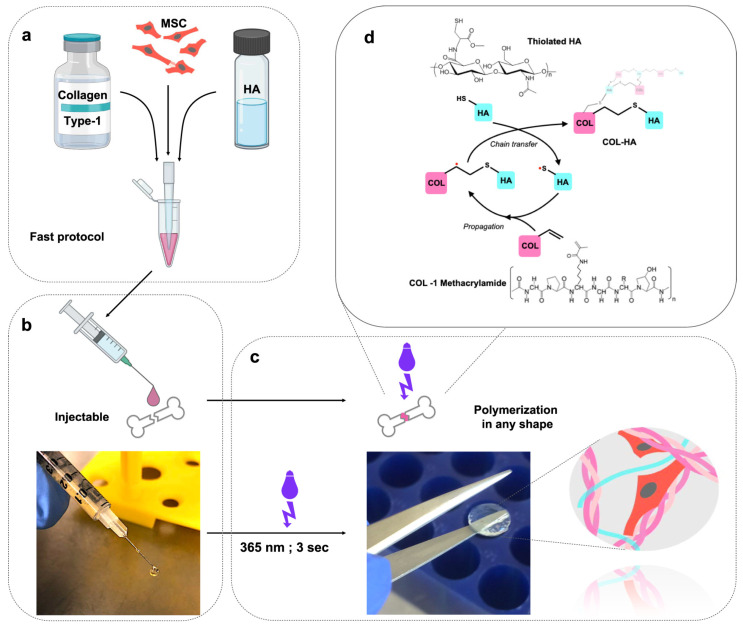
Fabrication of the COL-HA hydrogel. (**a**) MSCs are resuspended in the collagen–HA hydrogel. (**b**) The cell–hydrogel mix can be applied using a pipette or syringe. (**c**) The hydrogel can be polymerized on demand with a short UVA light pulse and maintains its shape in the crosslinked state. (**d**) Crosslinking of the biopolymer network through the radical mediated thiol–ene addition of thiolated HA to collagen methacrylamide [68].

The field of stem cell engineering and differentiation has witnessed substantial advancements, particularly through the development of advanced hydrogel platforms designed to enhance the therapeutic potential of stem cells. A notable innovation is a hydrogel composed of collagen I and hyaluronic acid (COL-HA), which is an effective medium for encapsulating mesenchymal stem cells (MSCs). This platform not only extends the secretory profile of MSCs but also boosts their ability to promote blood vessel growth, indicating that hydrogels capable of delivering biological signals can significantly enhance the therapeutic effects of MSCs (Figure 2) [68]. Additionally, the use of a bioactive hydrogel scaffold for the vascular differentiation of human embryonic stem cells (hESCs) has been proposed. By encapsulating hESCs in dextran-based hydrogels enriched with regulatory factors, this method effectively increases the expression of vascular markers, demonstrating the potential for applications in vascular tissue engineering [69].

Further studies have resulted in the creation of a dual-enzymatically crosslinked hyaluronic acid hydrogel, designed for the extended three-dimensional culturing of stem cells. This hydrogel is distinguished by its exceptional stability, tunable biodegradability, and substantial swelling ratio, underscoring its suitability for 3D cell culture and tissue engineering [76]. The development of a novel 3D hydrogel system for the ongoing culturing of adipose-derived mesenchymal stem cells also highlights its ability to sustain regenerative, non-senescent cell populations for extended periods without the need for subculturing, showcasing its potential in regenerative medicine [70].

Research into the interaction between mesenchymal stem cells and hyaluronan hydrogel scaffolds has shown that MSC encapsulation can alter the expression of genes associated with the extracellular matrix and cytokine production, especially when co-cultured with macrophages. This suggests that such encapsulation techniques may play a role in managing inflammatory responses and facilitating tissue regeneration [77]. The design of cell–matrix interactions in hyaluronic acid hydrogel scaffolds has been thoroughly reviewed, emphasizing the importance of incorporating bioactive, mechanical, and spatial signals to direct and control cell behavior in tissue engineering and regenerative medicine [78].

A thermally expandable hydrogel system, incorporating human dermal fibroblasts and coated with fibronectin and gelatin, has been developed to enable the rapid delivery of functional multi-layered cell sheets. This system facilitates the stable formation and efficient detachment of cell sheets, with a controlled presentation of cell-adhesive proteins, positioning it as a valuable tool in regenerative medicine [79]. Comparative studies on the differentiation of human pluripotent stem cells into vascular smooth muscle cells across various hydrogel-based cultures have shown that alginate hydrogels lead to cells with enhanced contractile phenotypes and the capacity to contribute to vasculature formation, highlighting the significance of hydrogel composition in stem cell differentiation [80]. 

The introduction of a chitosan/dextran-based hydrogel as a vehicle for delivering mesenchymal stem cells supports MSC growth and differentiation, suggesting its potential use in regenerative medicine and tissue engineering [81]. Moreover, the study of a polydopamine-coated gelatin–alginate hydrogel for modulating the adhesion and osteogenic differentiation of human adipose-derived stem cells has revealed the effectiveness of surface modifications in improving the regenerative capabilities of stem cells for clinical applications [82]. Together, these developments mark a significant step forward in the application of hydrogel technologies in stem cell engineering and differentiation, offering new avenues for repairing and regenerating damaged tissues.

## 7. Cartilage Regeneration

In regenerative medicine, with a particular focus on cartilage regeneration, significant progress has been achieved through the development of hydrogels and scaffolding techniques. These are meticulously designed to emulate the characteristics of natural tissues, thereby fostering the restoration of cartilage. A noteworthy advancement is the creation of a biomimetic hydrogel derived from the extracellular matrix of mesenchymal stem cells, which has shown promising results in inducing chondrogenesis and forming tissue akin to hyaline cartilage in vivo [83].

Addressing the challenge of mechanical stability in cartilage constructs, researchers have innovatively bonded a 3D-printed thermoplastic to a gelatin hydrogel. This technique strengthens the interface and increases the construct’s resistance to mechanical forces, making it particularly suitable for regenerative medicine, especially in load-bearing scenarios [7]. Another significant development is a hydrogel blend incorporating cellulose nanocrystals, gelatin methacrylate anhydride (GelMA), and hyaluronic acid–methacrylate anhydride (HAMA), enhanced with synthetic melanin nanoparticles and kartogenin. This formula not only facilitates cartilage restoration but also improves magnetic resonance imaging (MRI) contrast and enables the sustained release of therapeutic agents, allowing for non-invasive monitoring of the healing process [50].

**Figure 3:**
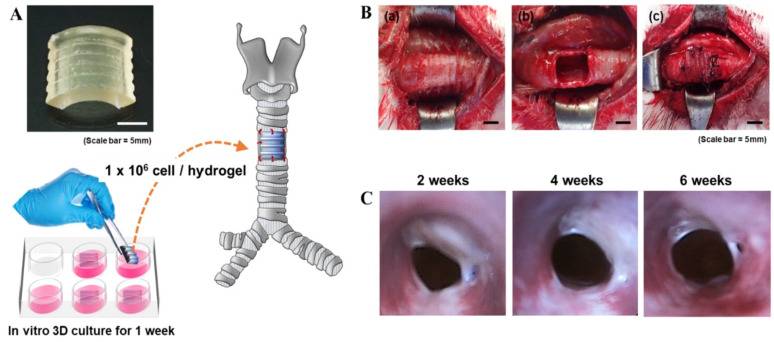
A schematic representation of chondrocyte-laden silk–GMA hydrogel transplantation and endoscopic observation of rabbit trachea for 6 weeks after transplantation. (**A**) The DLP-printed artificial trachea (10 × 10 × 2 mm, W × D × H) with chondrocytes from rabbit ears, cultured for 1 week. (**B**) (**a**,**b**) Removal of part of the trachea (10 × 10 mm). (**c**) Artificial trachea implantation. Scale bars represent 5 mm. (**C**) Endoscopy of trachea after transplantation at 2, 4, and 6 weeks. The transplanted chondrocyte-laden silk–GMA hydrogel showed that the internal diameter gradually increased after transplantation and the surrounding tissues grew into the surgical part of the trachea at 6 weeks after transplantation [10].

Further progress includes the use of silk fibroin, modified with glycidyl methacrylate for digital light processing (DLP) 3D printing, which has demonstrated potential in promoting chondrogenesis both in vitro and in vivo (Figure 3) [10]. An innovative injectable scaffold that combines collagen-coated polylactide microcarriers with a chitosan hydrogel has also been developed, aiming to enhance mechanical properties and promote chondrocyte proliferation and extracellular matrix production, showcasing its applicability in orthopedic procedures for cartilage regeneration [63].

A treatment strategy for osteoarthritis has emerged through the induction of chondrocyte autophagy via the sustained release of a leptin inhibitor from a thermosensitive hydrogel. This strategy utilizes a polymer blend (poly(D,L-lactide)-poly(ethylene glycol)-poly(D,L-lactide), PDLLA-PEG-PDLLA: PLEL), offering promising avenues for articular cartilage regeneration [64]. Additionally, a hydrogel/microparticle system capable of dual drug delivery—dispensing both melatonin and methylprednisolone—has been designed to enhance mesenchymal stem cell survival and boost proteoglycan content, crucial for cartilage tissue engineering [45].

Research comparing the cartilage repair efficacy of human umbilical cord Wharton’s jelly (HUCWJ) against that of hydrogels has indicated that HUCWJ exhibits superior cartilage repair capabilities [26]. The effectiveness of various cell types, including equine auricular cartilage progenitor cells, bone marrow-derived mesenchymal stem cells, and auricular chondrocytes, has been evaluated within gelatin methacryloyl-based hydrogels, focusing on their chondrogenic differentiation, matrix production, and mechanical properties [84]. Kappa-carrageenan hydrogels have been explored for their ability to deliver adipose tissue-derived stem cells for cartilage tissue repair, supporting cell viability, proliferation, and chondrogenic differentiation [85].

A thermo-reversible hydrogel composed of poly(N-isopropylacrylamide)-grafted methylcellulose has shown promise in articular cartilage regeneration by supporting cell viability and proliferation, as well as enhancing glycosaminoglycan synthesis [65]. The creation of a bioinspired supramolecular nanofiber hydrogel through the self-assembly of biphenyl–tripeptide has demonstrated excellent biocompatibility and supports cell adhesion, proliferation, chondrocyte spreading, and chondrogenic matrix secretion, marking its potential for tissue engineering [53].

Moreover, a photopolymerizable hydrogel combining methacrylated glycol chitosan and type I collagen has proven conducive to the survival, spread, and extension of encapsulated fibromodulin-reprogrammed cells. This environment facilitates the formation of myotubes in vitro without promoting undesired differentiation, showing gene expression profiles similar to those of satellite cells during myogenic differentiation [86]. These collective advancements signify a leap forward in hydrogel-based technologies for cartilage regeneration, opening new pathways for treating cartilage-related injuries and disorders.

## 8. Bone Regeneration

In the quest to improve bone regeneration, scientists have developed various innovative methods that employ hydrogels and tissue-engineered constructs. These methods are designed to replicate the complex structure and function of natural bone and cartilage tissues, thereby enhancing the healing processes of bone defects and injuries. One such approach involved embedding adipose-derived stem cells, which had been differentiated over periods of 7 and 14 days, within a three-dimensional collagen matrix situated on a microfluidic chip. This configuration enabled the precise evaluation of osteogenic markers, including alkaline phosphatase activity, calcium mineralization, and protein expression. These markers are essential for assessing the differentiation potential of the cells and monitoring their development over time, which is crucial for successful bone regeneration [57].

Furthermore, hybrid tissue-engineered constructs have been developed for the repair of osteochondral tissue. These constructs utilize cell-instructive modules composed of chondrogenic and osteogenic bioinks, along with allogeneic umbilical cord blood-derived mesenchymal stromal cells. The resulting constructs have shown the capability for both chondrogenic and osteogenic differentiation. This advancement offers a ready-to-use, single-surgery solution for the repair of osteochondral tissues, representing a significant leap forward in the field [87]. Another noteworthy development is the use of gelatin methacrylamide hydrogels embedded with multipotent stromal cells and cartilage-derived matrix particles. This approach supports matrix formation and degradation, thereby stimulating the creation of a cartilage template that can subsequently be remodeled into mineralized bone tissue, a process that mimics the natural development and healing of bone [88].

Additionally, a cutting-edge technique that combines the extrusion printing of a bone-biomimetic ceramic ink with the melt electrowriting of spatially organized polymeric microfibers has been introduced. This method enables the engineering of a reinforced interface between the bone and cartilage within an osteochondral plug. By enhancing the adhesion strength between the hydrogel and ceramic components, this technique supports osteogenesis and the deposition of a cartilage matrix in vitro [89]. 

Researchers have developed novel multicomponent organic–inorganic hydrogel composites incorporating whey protein isolates, gelatin, and alpha-tricalcium phosphate. These composites exhibited improved mechanical properties and supported osteoblast adhesion, spreading, and proliferation, highlighting their potential for bone tissue engineering [62]. Microcavitary hydrogels were created for cartilage tissue engineering, enabling cell colonies to expand into cavities for new tissue formation. This overcomes environmental limitations for new tissue generation within the hydrogel bulk [90].

**Figure 4:**
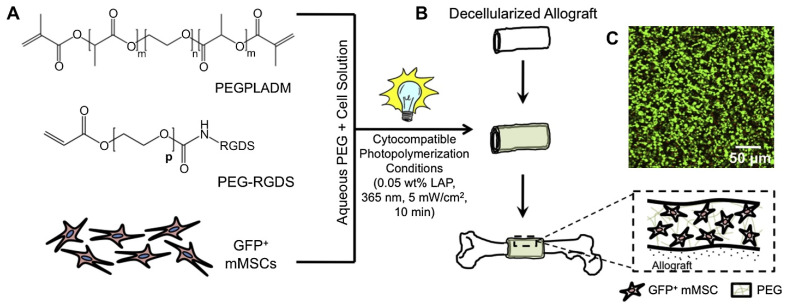
Scheme representing the tissue-engineered approach to enhance allograft healing. mMSCs were added to poly(ethylene glycol) macromer solutions (**A**) and custom molds were used to form hydrogel–cell constructs around decellularized allografts (e.g., tissue-engineered periosteum) (**B**). Encapsulated cells remained > 95% viable, as illustrated by the live/dead image (of GFP^−^ mMSCs; calcein AM (green = live cells) and ethidium homodimer (red = dead cells)) 24 h after encapsulation (**C**) [71].

Hydrolytically degradable hydrogels have been explored to transplant and localize mesenchymal stem cells on allograft surfaces, effectively creating a “tissue-engineered periosteum” (Figure 4). This resulted in increased graft vascularization, bone formation, and mechanical strength [71]. An N-cadherin-mimetic hydrogel was developed to enhance mesenchymal stem cell chondrogenesis by regulating cell metabolism, demonstrating its potential for tissue engineering and regenerative medicine by attenuating hypertrophy [55].

In vivo studies have demonstrated the osteogenic differentiation of human turbinate mesenchymal stem cells using an injectable in situ-forming hydrogel, providing a non-invasive alternative for bone tissue engineering with maintained cell viability and differentiation [91]. A gelatin-based gel incorporating platelet-rich plasma lysate was developed to mimic the role of a blood clot, facilitating bone reconstruction by supporting mesenchymal stem cell migration and differentiation into osteogenic cells [40].

**Figure 5:**
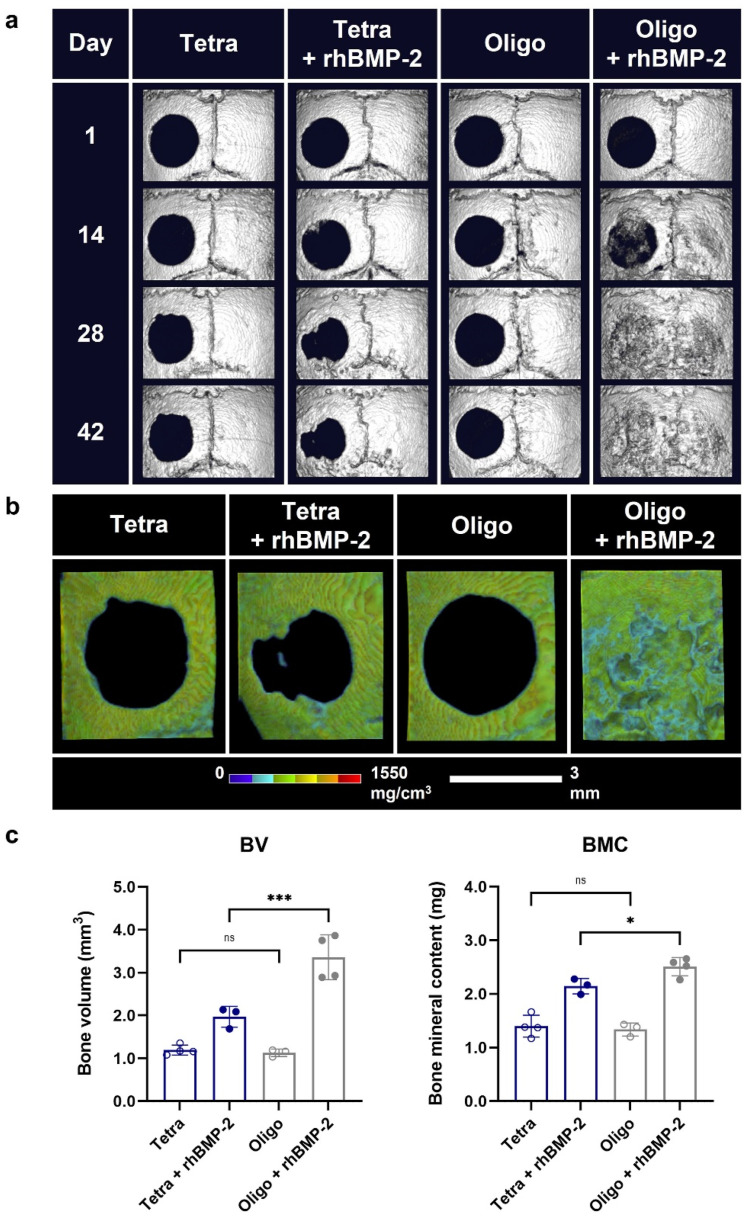
Comparison of bone regeneration between Tetra and Oligo gels loaded with and without rhBMP-2 in a 3 mm diameter mouse calvarial critical defect model over a 42-day period. (**a**) In vivo micro-CT representative images of defects at 1, 14, 28, and 42 days post operation. (**b**) Visualization of bone density of the defect site and surrounding area at 42 days post operation. The same trimming dimension and approximate imaging location is used across all samples. Scale bar: 3 mm. (**c**) Quantitative measurement of bone mineral content (mg) and bone volume (mm^3^) of the defect site and surrounding cortical bone regions at 42 days, as represented in (**b**). Data presented as mean ± SD, n = 3 or 4. Statistical analysis performed using two-way ANOVA and Tukey’s multiple comparison test. *p*-value * < 0.05, *** < 0.001, ns—not significant [35].

Tetra-PEG hydrogels (Tetra gels) were examined for bone repair, revealing that oligo-tetra-PEG gel (Oligo gel) underwent significant phase separation, showing higher hydrophobicity and enhanced growth factor release, which substantially improved the regeneration of critical-sized calvarial defects [35]. Figure 5 shows an in vivo cranial bone regeneration using rhBMP-2-loaded Tetra and Oligo gels [35]. A cytocompatible hydrogel made of gelatin–alginate was designed as a coating for beta-tricalcium phosphate scaffolds. This setup optimized crosslinkers to modulate the diffusion of proteins and support human adipose stem cell proliferation and osteogenic differentiation [92].

A photocrosslinkable methacrylated bone-derived ECM hydrogel was synthesized for bioprinting vascularized scaffolds, displaying tunable mechanical properties and promoting the rapid formation of vascular networks due to the pro-angiogenic molecules present in the bone ECM [93]. Composite scaffolds with a core–shell architecture combining a 3D-printed PLA core with a gelatin–chitosan hydrogel shell have also been studied for targeting bone regeneration, with tailored mechanical properties and an improved bioactive shell content [41].

Further research has led to the development of dental pulp stem cell (DPSC)-loaded conductive hydrogel microspheres. These microspheres, when integrated with a wireless electric generator, have been found to foster stemness and reduce heterogeneity among DPSCs through electric cues. This technique has shown promising results in promoting angiogenic behavior and anti-inflammatory macrophage polarization, making it a viable option for regenerating mandibular bone defects [73]. Additionally, injectable calcium phosphate scaffolds containing hydrogel fibers encapsulating human-induced pluripotent stem cells (hiPSCs), human embryonic stem cells (hESCs), and human umbilical cord mesenchymal stem cells (hUCMSCs) have demonstrated excellent cell viability, proliferation, and osteogenic differentiation. This innovation presents a promising strategy for enhancing bone regeneration across various applications, including dental, craniofacial, and orthopedic fields [72].

Comparative studies focusing on human auricular chondrocytes embedded in different hydrogel materials for cartilage tissue engineering have identified telopeptide collagen as a potentially effective clinical hydrogel. Despite this, there remains a need for further improvement in the gelling capabilities of synthetic peptides to achieve optimal cartilage regeneration outcomes [47].

## 9. Neural and Spinal Cord Regeneration

In the pursuit of advancing neural and spinal cord regeneration, recent studies have delved into hydrogel-based methodologies aimed at fostering environments conducive to the growth, differentiation, and repair of neural stem cells (NSCs). This research underscores the importance of the physical properties of hydrogels, such as their stiffness, in influencing NSC behavior. Notably, hydrogels with a softer modulus, akin to the natural softness of brain tissue, have been shown to enhance the expression of neuronal markers, indicating an optimal environment for NSC differentiation within three-dimensional scaffolds like alginate hydrogels [46].

Utilizing phenyl azide photochemistry and optical fiber technology, researchers have created a 3D hydrogel scaffold tailored for spinal cord injury repair. This approach, which includes a co-culture assay, focuses on localizing adhesive proteins to the inner surfaces of nerve guidance conduits, thereby improving NSC adhesion and survival. Such advancements have demonstrated potential in promoting locomotion recovery and guiding neuron tissue formation post implantation [94]. Moreover, a hierarchical hybrid gelatin methacrylate–microcapsule hydrogel embedding neurotrophin-3-loaded microcapsules has been developed. This hydrogel establishes an anisotropic structure with patterned neurotrophin (NT-3) distribution, showcasing the possibility of constructing various biomimetic soft scaffolds to support spinal cord regeneration [95].

Porous hydrogel tubes, created through a two-step polymerization process, have been designed to guide axon growth across spinal cord injury sites. These tubes not only integrate well with host tissues but also mitigate glial scarring and bolster axon growth and functional recovery, highlighting their potential use in various tissue repair contexts [96]. Gelatin-based hydrogels have been employed for the release of vascular endothelial growth factor (VEGF) in peripheral nerve tissue engineering, enhancing neurite outgrowth and affirming these hydrogels’ suitability for neural tissue engineering applications [42].

The application of hydrogels extends to monitoring and guiding tissue regeneration in stroke and neurological disorder models. Diamagnetic chemical exchange saturation transfer imaging has been utilized to track the in vivo distribution of extracellular matrix hydrogel implants within stroke cavities, providing valuable insights into their temporal evolution [67]. A novel approach that integrates human marrow-isolated adult multilineage-inducible stem cells with pharmacologically active microcarriers in a non-toxic silanized-hydroxypropyl methylcellulose (Si-HPMC) hydrogel aims to direct neural/neuronal differentiation and augment the therapeutic secretome, offering a promising treatment pathway for neurological disorders [97].

Electrospun membranes alongside visible light-crosslinked gelatin hydrogels have been devised for nerve guidance conduits, creating microgrooved surface patterns that effectively foster axon regeneration and functional recovery, presenting an alternative to autogenous nerve grafts [51]. In intervertebral disc regeneration, injectable chitosan nanocellulose hydrogels encapsulating stem cells have shown significant promise in alleviating degeneration and stabilizing injured discs in an ovine model [52].

For the bioprinting of neural tissue constructs, conductive collagen/polypyrrole-b-polycaprolactone hydrogels have been introduced. These hydrogels enhance neural differentiation and tissue regeneration, combining improved printability with biocompatibility, thus being suitable for repairing damaged neural tissues and facilitating drug testing [98]. Additionally, de novo self-assembling peptide hydrogels (SAPHs) have been explored as cell carriers and scaffolds for nucleus pulposus tissue engineering, demonstrating promising outcomes in gene upregulation, cell viability, and the synthesis of key extracellular matrix components [99].

The mechanical stability of RADA16 peptide hydrogels has been enhanced by blending them with a photocrosslinkable triblock copolymer, resulting in a hybrid hydrogel with improved mechanical properties appropriate for spinal cord injury repair applications [60]. The development of polymers with a spiral scaffold design and cell-laden fibrin hydrogels for 3D bioprinting of the urethra showcases biomimetic mechanical properties and a supportive environment for cell growth, laying an in vitro foundation for future progress in urethral reconstruction within regenerative medicine using dual-autologous cells in fibrin hydrogels [58]. These diverse approaches underscore the continuous innovation and potential of hydrogel-based systems in the field of neural and spinal cord regeneration, offering new avenues for effective treatment and recovery strategies for nervous system injuries and disorders.

## 10. Soft Tissue and Organ Regeneration

Recent studies in soft tissue and organ regeneration have leveraged hydrogels and biocomposite materials to mimic the natural extracellular matrix (ECM), thereby promoting the growth and differentiation of stem cells into functional tissues. Researchers have developed a hydrogel platform for assembling human stem cell-derived islet cells and endothelial cells into islet organoids. These organoids are capable of expressing beta cell markers and secreting insulin in response to glucose, serving as a promising model for studying the islet microenvironment and advancing tissue engineering [100].

The mechanical properties of hyaluronic acid (HA) hydrogels, such as their stiffness, have been identified as a key factor in the repair of muscle injuries. Specifically, hydrogels with medium stiffness have been shown to support sustained regenerative outcomes and mitigate chronic inflammatory responses, emphasizing the critical role of hydrogel stiffness in muscle tissue repair [101]. Another innovative approach has been the creation of uniform-sized 3D hepatocyte spheroids within a hybrid hydrogel comprising alginate and ECM molecules. This strategy enhances hepatic marker expression and secretory functions, demonstrating its broad application potential in tissue engineering and regenerative medicine [102].

Collagen type I microspheres have been fabricated to efficiently differentiate pluripotent stem cells into hepatocyte-like cells, thus forming pre-vascularized liver tissue. This technique represents a significant step forward in stem cell-based liver tissue fabrication for applications in regenerative medicine, drug screening, and liver modeling [103]. In cardiac tissue, functional cardiac tissue microspheres have been generated through the direct differentiation of hydrogel-encapsulated human-induced pluripotent stem cells (hiPSCs), achieving high reproducibility and functionality of cardiomyocytes. This development is pivotal for biomanufacturing, drug screening, and regenerative therapies [104].

A heparin–hyaluronic acid hydrogel, crosslinked through thiolated heparin and methacrylated hyaluronic acid, has been shown to support 3D cellular activities, demonstrating its effectiveness for 3D cell culture engineering and its suitability for stem cell therapy and tissue engineering without requiring additional modifications [105]. Pre-formed alginate hydrogel microtubes have been used to facilitate direct epithelial–mesenchymal cell interactions for 3D salivary gland cell organization. This method supports heterotypic cell–cell interactions and organoid formation, with potential applications in regenerative medicine and tissue morphogenesis [106].

In engineering hepatic tissues, a dual approach has been employed to control the hydrogels’ physicomechanical properties and microarchitecture, optimizing hepatic function within an in vivo liver injury model [107]. Gelatin-based biocomposite films, loaded with an epithelial cell growth factor cocktail, have been designed to mimic the ECM for regenerating respiratory epithelia from stem cells, with applications in regenerative medicine and the in vitro modeling of barrier tissues [43].

Silk fibroin hydrogels have been explored for their application in oral and craniomaxillofacial tissue regeneration, highlighting their biocompatibility, safety, and potential for combination with other materials for high-quality tissue regeneration [108]. The use of electrospinning and hydrogel composites for engineering and regenerating soft tissues has been discussed, with electrospun nanofiber scaffolds showing morphological similarities to the natural ECM and enhancing biological performance for complex tissue engineering [109].

Carrageenan hydrogel has been presented as a scaffold for human skin-derived multipotent stromal cells, demonstrating its potential as a cell carrier for tissue engineering and promoting recovery in models of full-thickness skin wounds [110]. A supercritical carbon dioxide and ethanol co-solvent decellularization method for the heart ECM preserved the ECM’s structure and angiogenic proteins when tested on rat heart tissues for improved angiogenesis [111].

Patterned GelMA-PEGDA hydrogels, grafted with RGD peptides and using molding technology, have been developed to construct epidermal models with rete ridges, enhancing cell adhesion and maintaining the epidermal stem cell niche [112]. A 3D-bioprinted microchanneled gelatin hydrogel for cardiac tissue engineering has been shown to promote hMSC myocardial commitment and support cardiomyocyte functionality, affecting cell alignment and differentiation [44].

Hydrogel carrier systems utilizing human hair-derived keratins have been explored for addressing volumetric muscle loss by delivering growth factors and muscle progenitor cells, with tunable degradation and cell support [113]. Chitosan hydrogels, as carriers for brown adipose-derived stem cells, have been shown to enhance cardiac differentiation and survival post myocardial infarction, leveraging collagen synthesis enhancement for cardiac tissue engineering [114]. A biodegradable and antioxidant DNA hydrogel, incorporated with interleukin-33, has been utilized as a cytokine delivery system for diabetic wound healing, facilitating inflammation resolution and wound closure [115].

Heparin hydrogel microwells have been developed for primary hepatocyte cultivation, testing various surface modifications to enhance hepatic phenotype maintenance, highlighting the benefits of all-heparin gel microwells for liver tissue engineering [116]. Injectable hyaluronic acid-derivative hydrogels, modified with RGD and combined with fibrinogen, have been tested in spinal cord injury repair, facilitating cell adhesion and proliferation for neural tissue engineering [117]. Lastly, interpenetrating network hydrogels of hyaluronic acid and fibrin, prepared in situ, enhance mechanical properties and cell proliferation, presenting a promising scaffold for extracellular matrix-based tissue regeneration [118].

These advancements reflect significant progress in developing hydrogel-based solutions for soft tissue and organ regeneration, offering new strategies for restoring function and improving the quality of life for individuals with tissue damage or organ failure.

## 11. Other Tissue Engineering Applications

In regenerative medicine, remarkable progress has been made with the introduction of innovative materials designed to facilitate tissue regeneration across various applications. One breakthrough is the development of a fully synthetic, thermoresponsive, and injectable hydrogel that adheres to the site of administration, thereby promoting the growth of new blood vessels and the integration of host cells for the repair of both soft and hard tissues [21]. Similarly, hydrogel scaffolds derived from blood plasma cryoprecipitate and collagen, prepared via enzymatic hydrolysis, have demonstrated excellent biocompatibility and the ability to support the three-dimensional expansion of adipose tissue stem cells, positioning them as ideal cell carriers in regenerative therapies [38].

A tunable alginate hydrogel scaffold system has been devised specifically for modeling growth plate cartilage. This system facilitates the precise manipulation of chondrocyte differentiation and spatial organization, closely mimicking natural growth patterns, which could significantly impact personalized treatments in regenerative medicine [37]. Furthermore, research into the effects of scaffold porosity and oxygenation has revealed that increased porosity notably enhances oxygen delivery, cell viability, and osteogenic differentiation within 3D-printed hydrogel constructs, highlighting its critical role in bone tissue engineering [25].

An exploration into the modification of cysteine thiol groups on keratin for tunable hydrogel erosion aims to control disulfide crosslinking. This strategy seeks to develop hydrogels with adjustable erosion rates for precise drug delivery, without compromising cellular compatibility, thus expanding their application in tissue engineering [23]. The creation of a hybrid hydrogel combining collagen and a PEG-derived polymer offers improved mechanical strength and viscoelastic properties, maintaining an optimal balance between biodegradability and biocompatibility. Such characteristics make it a superb scaffold for tissue defect repair [39].

The use of extracellular matrix hydrogels from the porcine urinary bladder has highlighted their biocompatibility and biodegradability, which significantly enhance cell colonization and their integration into host tissues. This strategy is designed to effectively tackle the challenges associated with repairing irregularly shaped defects [9]. Additionally, the development of a tissue-adhesive hydrogel through recombinant tyrosinase-mediated crosslinking has been optimized for rapid gelation, enhanced physical properties, and strong adhesive qualities, enabling its use as an injectable and sprayable solution across various applications in regenerative medicine [119].

The introduction of a biodegradable, thermosensitive, and injectable hydrogel made from carboxymethyl chitin (CMCH) marks another advancement. This hydrogel exhibits quick gelation at body temperature, with tunable gelation times, and supports cell survival and proliferation both in vitro and in vivo [28]. Moreover, a platform for the 3D dynamic mechanical stretching of arrayed biomaterial constructs has enabled the systematic study of mechanobiological effects on cell fate and function, especially relevant in the context of connective and cardiovascular tissue engineering [5].

Research into various crosslinking methods for chitosan/gelatin/egg-white composite hydrogels has identified TPP crosslinking as particularly effective in enhancing their physical and biological properties, fostering new vessel growth and angiogenesis [17]. The biocompatibility and angiogenic potential of extracellular matrix hydrogels from the porcine urinary bladder, biofunctionalized with the LL-37 peptide, have been evaluated, showing the capacity of these hydrogels to induce angiogenesis in vivo without adversely affecting cell proliferation, thus demonstrating their suitability for tissue regeneration therapies [49].

Innovative hydrogel formulations continue to emerge, such as an injectable hydrogel based on dialdehyde galactomannan and N-succinyl chitosan, employing Schiff base crosslinking to create a quick-setting, highly injectable scaffold with a macroporous architecture, suitable for cell culture and soft tissue engineering [66]. The design of an injectable hyaluronic acid-based hydrogel enhanced with biogenically synthesized silver nanoparticles and multi-walled carbon nanotubes shows potential for antibacterial applications in treating wound cavities or bone defects [61].

The engineering of a thixotropic supramolecular gelatin-based hydrogel for cell transplantation has demonstrated its injectability and capacity to support cell adhesion, spreading, and migration, offering a promising method for enhancing graft survival in regenerative medicine [120]. A 3D-printable hyaluronic acid-based hydrogel has been developed for use as a bioink in tissue engineering, characterized by excellent rheological properties, biocompatibility, and printability [16]. Additionally, the combination of mesenchymal stem cells with a nano-polypeptide hydrogel for engineering blood vessels through 3D-bioprinting technology addresses the limitations of current vascular grafts, signaling a way forward in tissue engineering [121].

Hybrid hydrogels incorporating carbon nanotubes have been created to feature adjustable electrical and mechanical properties. This development significantly broadens the scope of hydrogel technology in regenerative medicine, tissue engineering, and drug delivery devices, promising enhanced functionality and adaptability for a wide range of biomedical applications [122]. Additionally, a chitosan and hyaluronic acid hydrogel has been specially tailored for bioprinting purposes. This hydrogel aims to replicate the natural environment of the extracellular matrix, as demonstrated by thorough analyses of its morphology, cytotoxicity, and cell viability. Such findings underscore its potential for widespread use in tissue engineering and regenerative medicine (TERM) applications, indicating a step forward in the development of biocompatible and effective scaffolding materials [123].

Research into the viscoelastic properties of hydrogels has uncovered that matrices that relax more quickly can significantly enhance mesenchymal stem cell (MSC) spheroid migration and fusion. This observation opens new avenues for tissue engineering strategies that utilize spheroids, providing insights into how the physical properties of hydrogels can be optimized to support advanced cellular behaviors and tissue formation processes [124]. The creation and use of a composite hydrogel consisting of polyacrylamide, graphene oxide, gelatin, and sodium alginate has been shown to accelerate peripheral nerve regeneration. This hydrogel supports Schwann cell attachment and proliferation, highlighting its potential in nerve tissue engineering and restoring function following nerve injuries [48].

A novel approach involving injectable hydrogel microspheres that incorporate bioactive factors derived from a decellularized dental pulp matrix aims to recreate a specific three-dimensional microenvironment. This environment is conducive to enhancing dental pulp stem cell differentiation and facilitating the regeneration of the pulp–dentin complex, indicating a promising direction for dental regenerative therapies and endodontic applications [125]. Furthermore, a predictive model for the degradation of gelatin–PEG composite hydrogels has been developed. This model aims to control the degradation rate and improve the mechanical properties of the hydrogel, thereby supporting the growth of human mesenchymal stem cells. This advancement holds potential for applications in drug delivery and tissue engineering, highlighting the importance of material properties in the design of regenerative scaffolds [6].

The integration of photobleaching and thiol–Michael addition reactions to model thiol–acrylate photopolymerization kinetics represents a significant advancement in understanding and optimizing the mechanical properties of hydrogels. This knowledge is crucial for designing scaffolds that meet the specific needs of regenerative medicine, offering customized solutions for tissue repair and regeneration [11]. Furthermore, the development of tunable polysaccharide hydrogel blends based on dextran, chitosan, methylcellulose, and agarose for neural repair emphasizes the significance of softer, more positively charged hydrogels. These hydrogels are particularly effective in enhancing neuron attachment and neurite extension, suggesting their utility as scaffolds for the regeneration of the central nervous system. This insight further illustrates the potential of hydrogels to provide targeted and specialized support for nerve repair and neuroregenerative strategies [126].

## 12. Collective Outcomes

Hydrogels have become a cornerstone in tissue engineering, especially recognized for their capacity to closely mimic the intricate biological processes crucial for the maturation and development of oocytes. This is largely due to their modifiable mechanical attributes and their inherent versatility, highlighting their indispensable role in this specialized field [1,13]. The integration of bio-adhesive motifs and the incorporation of macropores into these hydrogels have notably advanced their functionality, significantly bolstering their ability to support cell adhesion, proliferation, and the deposition of the extracellular matrix. These enhancements signal a leap towards replicating the complexities of biological tissues artificially [29,31].

Beyond just their composition, hydrogels have seen technological enhancements in biomechanical tuning, cryopreservation techniques, achieving uniform cell seeding, the customization of scaffolds with high resolution, and achieving superior mechanical properties. Such innovations have elevated hydrogels to a prominent position in creating scaffolds that precisely mirror the properties of natural tissues, marking a significant step in the innovative use of hydrogels within tissue engineering [4,20,32,33,75].

Hydrogels have showcased remarkable efficacy in supporting regeneration across both soft and hard tissue types, evidenced by their success in numerous preclinical and clinical trials, affirming their versatility and effectiveness [21]. Their exceptional biocompatibility plays a pivotal role in facilitating three-dimensional growth and enhancing mesenchymal stem cell functionalities, crucial aspects for augmenting therapeutic outcomes [38,68,69].

The adaptability of hydrogels is further exemplified by their customizability and tunability, enabling the modeling of patient-specific tissues and controlled drug delivery via adjustable erosion rates, showcasing their ability to cater to a wide array of medical requirements [23,37]. Their non-toxic nature, rapid gelation abilities, robust adhesive strength, and minimal immune responses further solidify their suitability and efficacy for various medical applications [9,119].

Ongoing innovation in tissue engineering is highlighted through strategies employing 3D dynamic mechanical stimulation and optimizing crosslinkers for angiogenesis, showcasing the cutting-edge role of hydrogels in contemporary research [5,17]. Hydrogels have proven instrumental in enabling long-term three-dimensional cell culture engineering, differentiating cells into specific cell types, and fostering chondrogenesis, thus showing their potential for use in targeted tissue regeneration and cartilage repair [64,70,80,82,83].

Moreover, enhancements in hydrogels’ mechanical properties and the development of non-invasive monitoring techniques, like MRI, have improved their translational potential in clinical settings [7,50]. In the domains of drug delivery and tissue engineering, hydrogels have been successfully utilized to achieve sustained dual drug release and maintain cell viability and differentiation over extended periods, affirming their role as versatile scaffolding materials [45,65,85].

The capability of hydrogels to facilitate in vivo tissue remodeling and their innovative applications in microfluidic settings and hybrid biofabrication underline their support of complex tissue regeneration processes and their versatility in biomedical applications [57,87,88]. Material integration improvements, stability enhancements, and the facilitation of new tissue formation highlights the potential of hydrogels in creating durable implants and addressing intricate challenges in regenerative medicine [62,71,89,90].

Hydrogels have also demonstrated their ability to promote osteogenic and chondrogenic differentiation, contributing to bone health and complete bone defect regeneration and providing mechanical support for osteogenic factors, showcasing their effectiveness in bone recovery processes [35,40,92]. Their role in supporting vascularization and constructing immuno-angiogenic niches highlights their multifaceted functionality in regenerative strategies [73,93].

In models of spinal cord injury, hydrogels have significantly enhanced neuronal marker expression, reduced glial scarring, and facilitated neurite outgrowth, contributing greatly to the repair of the central nervous system and peripheral nerve repair [42,46,94,95,96]. Advances in hydrogel printability and biocompatibility have opened up new possibilities in bioprinting neural tissue constructs, enriching the landscape of neural tissue engineering [98].

Their effectiveness in facilitating the imaging of extracellular matrix hydrogel distribution, supporting functional islet organoids, and aiding muscle recovery underscores the essential role of hydrogels in generating functional tissues and organoids, signaling new advancements in wound healing, cardiac tissue engineering, and the remediation of volumetric muscle loss [44,67,100,101,102,110,113]. The exploration of innovative hydrogel formulations, such as silk protein hydrogels and all-heparin gel microwells, reflects continuous innovation, offering fresh avenues for the development of advanced biomaterials [108,116]. This comprehensive analysis displays the transformative impact of hydrogels across various aspects of tissue engineering and regenerative medicine, highlighting their significant potential for future biomedical advancements.

## 13. Limitations

The quest for optimal bioinks represents a crucial area of research within tissue engineering, focusing on identifying materials that satisfy a comprehensive set of criteria. These criteria include enhancing cell attachment, ensuring effective printability, and facilitating cell migration. This endeavor emphasizes the current dependency on certain materials, such as gelatin, revealing a notable gap in the diversity of hydrogels tailored for specific applications like cartilage repair, as demonstrated in several studies [3,15,26,48,123].

Despite significant progress in the field, a prominent challenge persists in refining hydrogels to accurately mimic the mechanical properties of natural tissues. These properties comprise strength, electrical conductivity, and long-term stability. Additionally, the inherent viscoelastic nature of hydrogels introduces complexities related to aging and creep, where continuous stress or strain can lead to time-dependent deformation. This behavior may compromise the hydrogel’s ability to provide sustained support for cell attachment, migration, and proliferation—crucial for effective tissue repair and regeneration. Addressing these challenges requires a well-designed approach to balance mechanical characteristics with biological functionalities to suit specific cell types and their functions, while also ensuring the longevity of the materials in clinical applications [22,33,41,58,66].

Another critical hurdle involves precisely controlling stem cell behavior, including their proliferation, differentiation, and integration with the body’s tissues—key factors in the processes of healing and regeneration. This challenge extends to promoting cell mobility and proliferation, emulating signals from the natural extracellular matrix, and seamlessly integrating with the body’s tissues. Managing immune responses to and enhancing the bioactivity of hydrogel-based implants and grafts are also vital for their success in regenerative therapies [43,48,77,78,81,124].

Mimicking the complex structure and functionality of natural tissues, such as cartilage, presents a formidable challenge. It is not only about replicating the mechanical attributes of tissues but also maintaining cell viability and ensuring that the engineered structures functionally integrate with existing tissues in the body [58].

Efforts are continually being made to advance hydrogels toward achieving sophisticated capabilities in controlled and targeted drug delivery. This initiative aims to develop systems capable of dual-phase drug release or precise dispensation of growth factors and other therapeutic molecules. The objective is to enhance treatment efficacy while minimizing adverse effects, thereby improving patient outcomes in regenerative medicine [45,60]. This ongoing research and development of hydrogel technology reflects a commitment to overcoming current limitations, paving the way for innovative solutions in tissue engineering and regenerative medicine.

## 14. Future Directions

In the field of materials science, specifically within the domain of developing innovative hydrogel formulations, there is a focused effort aimed at enhancing the mechanical, chemical, and biological properties of these materials. This enhancement is crucial for improving their integration and efficacy in tissue regeneration. Innovations include the introduction of novel crosslinking methods and the incorporation of bioactive additives, which are designed to closely mimic the biochemical and mechanical characteristics of natural tissues, as demonstrated in various studies [15,29,78,120]. Concurrently, advancements in bioink and 3D printing technologies are directed at overcoming existing challenges related to bioink printability and mechanical stability. These innovations are pivotal in supporting complex tissue formation and facilitating cell migration, signifying a significant progression towards more sophisticated bioprinting applications [79,123].

In biomedical engineering, particularly as it applies to clinical practices, there is an increasing focus on regenerative and personalized medicine. This focus involves customizing hydrogel technologies to meet the unique needs of individual patients, employing 3D bioprinting to fabricate patient-specific scaffolds, and integrating patient-derived cells for more personalized treatment approaches [23,37,79]. Moreover, venturing into whole-organ engineering emerges as a promising direction for advancing the repair and replacement of organs, especially in addressing challenges related to scaffold vascularization and integration with host tissues for improved clinical outcomes [24].

The evolution of hydrogel technologies is further propelled by the integration of advanced manufacturing techniques and computational modeling. These methodologies are instrumental in expediting the design, prototyping, and testing of new hydrogel formulations. The application of breakthroughs in 3D bioprinting and microfluidics is particularly noteworthy for constructing complex tissue constructs, marking a significant advancement in the capabilities of hydrogel materials [6,11].

At the heart of these advancements lies interdisciplinary collaboration, which bridges the gap between materials science, bioengineering, chemistry, and clinical research. Such collaboration is essential for developing smarter, more responsive hydrogel systems capable of effectively mimicking natural tissue properties, enhancing mechanical strength, promoting cellular interactions, and supporting tissue integration. This collaborative effort underscores the innovation in crosslinking methods and materials science, highlighting the significance of interdisciplinary expertise in expanding the potential applications of hydrogel technologies [5,17,48].

Transitioning from laboratory research to clinical applications necessitates a deeper focus on in vivo studies to comprehensively understand the long-term behavior, efficacy, and safety of hydrogels in practical scenarios. Addressing regulatory challenges associated with the clinical translation of hydrogel-based therapies is paramount for ensuring that these innovative materials can be safely and effectively used in medical treatments [6,11].

Additionally, exploring hydrogels in combination with other therapeutic modalities, such as gene therapy and immunomodulation, offers a multifaceted approach to treatment. This strategy utilizes the unique properties of hydrogels to deliver a comprehensive therapeutic impact, showcasing a forward-thinking methodology that combines various treatment strategies for enhanced patient outcomes. This integrated approach emphasizes the dynamic and evolving nature of hydrogel research, highlighting its transformative potential in the fields of biomedical engineering and regenerative medicine.

**Figure 6:**
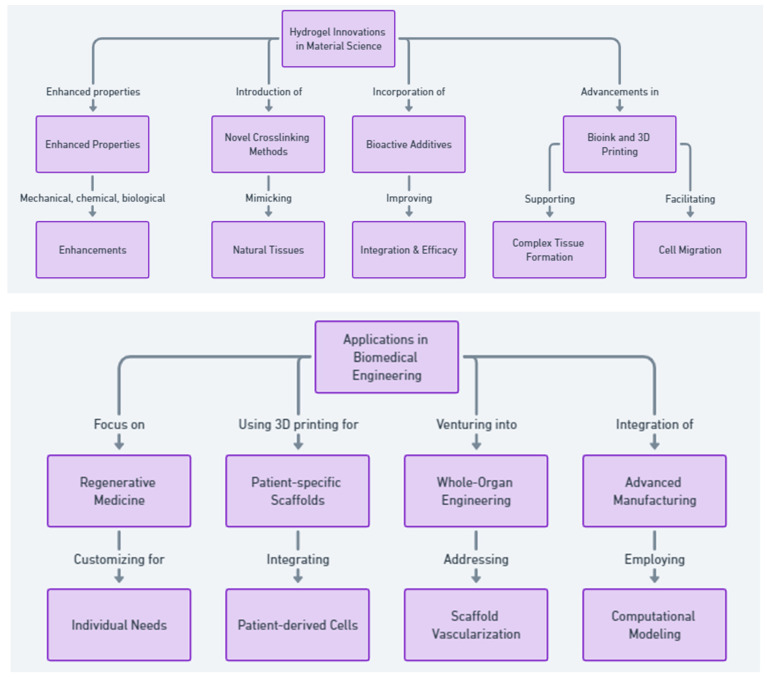
Innovations and applications of hydrogel-based materials in regenerative medicine and related fields.

Figure 6 illustrates the comprehensive pathway of innovations and applications of hydrogel-based materials in regenerative medicine, biomaterials, bone regeneration, and tissue engineering. It highlights the progression from innovations in materials science—such as enhanced properties, novel crosslinking methods, and the incorporation of bioactive additives—to advancements in bioink and 3D printing. The flowchart details applications in biomedical engineering, including regenerative and personalized medicine, patient-specific scaffolds, whole-organ engineering, and the integration of advanced manufacturing techniques. It also emphasizes interdisciplinary collaboration for developing smarter, more responsive hydrogel systems, and the transition to clinical applications, addressing in vivo studies and regulatory challenges. Furthermore, it showcases the combination of hydrogels with other therapeutic modalities for a comprehensive therapeutic impact, marking a significant leap in the field’s ability to support complex tissue regeneration and personalized medicine approaches.

## 15. Conclusions

Hydrogels have transformed the landscape of tissue engineering and regenerative medicine, serving as a cornerstone for innovations across a broad spectrum of biomedical applications. Their unique ability to mimic the mechanical, chemical, and biological properties of natural tissues has made them indispensable in the development of lifelike artificial tissues and organs. The progress in hydrogel technology, from enhanced material compositions to advanced fabrication techniques, features a significant leap forward in the ability to support complex tissue regeneration and personalized medicine approaches. Moreover, the integration of hydrogels with other therapeutic modalities opens new avenues for comprehensive treatment strategies. Despite facing challenges in mimicking the exact natural tissue properties and controlling stem cell behavior, the ongoing research and interdisciplinary collaborations promise to overcome these hurdles, paving the way for hydrogels to play a central role in future biomedical breakthroughs.
